# The SPFH Protein Superfamily in Fungi: Impact on Mitochondrial Function and Implications in Virulence

**DOI:** 10.3390/microorganisms9112287

**Published:** 2021-11-03

**Authors:** Marienela Y. Heredia, Jason M. Rauceo

**Affiliations:** Department of Sciences, John Jay College of the City University of New York, New York, NY 10019, USA; heredia3@wisc.edu

**Keywords:** SPFH, prohibitin, stomatin, mitochondria, fungi

## Abstract

Integral membrane proteins from the ancient SPFH (stomatin, prohibitin, flotillin, HflK/HflC) protein superfamily are found in nearly all living organisms. Mammalian SPFH proteins are primarily associated with mitochondrial functions but also coordinate key processes such as ion transport, signaling, and mechanosensation. In addition, SPFH proteins are required for virulence in parasites. While mitochondrial functions of SPFH proteins are conserved in fungi, recent evidence has uncovered additional roles for SPFH proteins in filamentation and stress signaling. Inhibitors that target SPFH proteins have been successfully used in cancer and inflammation treatment. Thus, SPFH proteins may serve as a potential target for novel antifungal drug development. This review article surveys SPFH function in various fungal species with a special focus on the most common human fungal pathogen, *Candida albicans*.

## 1. Introduction

The SPFH protein family is present in all domains of life. Proteins of this family are characterized by a conserved SPFH domain and diverge highly at their N- and C-terminal regions [1,2,3,4]. Furthermore, the distribution of SPFH proteins across species varies [1,2,4]. Proteomic and cellular analyses identified SPFH proteins in various cellular membranes, such as the inner mitochondrial membrane and plasma membrane [5,6,7,8,9,10,11,12]. SPFH proteins also localize to the endoplasmic reticulum and lysosome/vacuole [5,13,14]. Biochemical events dependent on SPFH proteins include palmitoylation and oligomerization and have supported a hypothesis that SPFH proteins are membrane scaffolds [15]. In vitro biochemical results showed that human stomatin protein binds directly to cholesterol and actin mainly through key amino acid sequences in the C-terminus [16]. Moreover, sequences in the SPFH domain are required for SPFH protein homo-oligomerization [16,17]. However, the details underlying the molecular function of SPFH proteins are limited.

SPFH proteins in mammals carry diverse functions. Prohibitin 1 (PHB1) and prohibitin 2 (PHB2) regulate mitophagy, or the removal of damaged mitochondria [18,19]. Stomatin-like protein 2 (SLP2) is required for respiratory chain complex assembly and mitochondrial translation [15,20,21,22]. Additional physiological roles associated with mammalian and nematode SPFH proteins include signaling, mechanosensation, and ion transport [23,24,25,26]. Importantly, disruption of SPFH protein function has been linked to several life-threatening conditions in humans, such as cancer, Alzheimer’s disease, kidney disease, and cardiac disease [27,28,29].

Several parasites require SPFH proteins for virulence. In the malaria-causing parasite, *Plasmodium berghei*, the prohibitin-like protein PHBL maintains mitochondrial membrane potential, and *phbl-* mutants failed to colonize their mosquito host [30]. Protozoan parasites from the genus *Leishmania* colonize vertebrate macrophages, causing chronic and debilitating skin diseases [31]. In *L. major*, mitochondrial prohibitin 1 and prohibitin 2 mediate survival in response to macrophage-induced oxidative stress [31]. Lastly, in the parasite, *Trypanosoma brucei*, the causative agent of African sleeping sickness, prohibitins are required for mitochondrial translation and maintaining mitochondrial membrane potential [32]. The diverse functions of SPFH proteins and their contributions to human disease emphasize the biological and clinical relevance of studying this protein superfamily.

## 2. SPFH Protein Function in Non-Pathogenic Fungi

Most of the knowledge for SPFH protein function in fungi has been acquired from experiments using non-pathogenic species [28,33,34,35,36,37,38]. In those studies, the mitochondrion has emerged as a focal point for understanding SPFH function. The mitochondrion is a specialized organelle found in eukaryotes and serves as the master regulator of metabolism, generating ATP via oxidative phosphorylation. Mitochondria also control key physiological activities, such as lipid synthesis and trafficking, aging, reactive oxygen species production, apoptosis, and cellular signaling.

In the baker’s yeast *Saccharomyces cerevisiae*, the sole SPFH proteins, prohibitin 1 and prohibitin 2 (Phb1, Phb2), form ring-shaped complexes within the inner mitochondrial membrane and are associated with several mitochondrial functions [9]. Phb1 and Phb2 interact with Mdm33 to regulate mitochondrial ultrastructure and shape [34]. In addition, Phb1 and Phb2 interact with the chaperones Atp10 and Atp23 to assist formation of F_1_F_o_-ATP synthase [37]. Depletion of prohibitins reduces yeast life span and is characterized by abnormal mitochondrial structure and delayed mitochondrial segregation to budding daughter cells [36,39,40].

**Figure 1:**
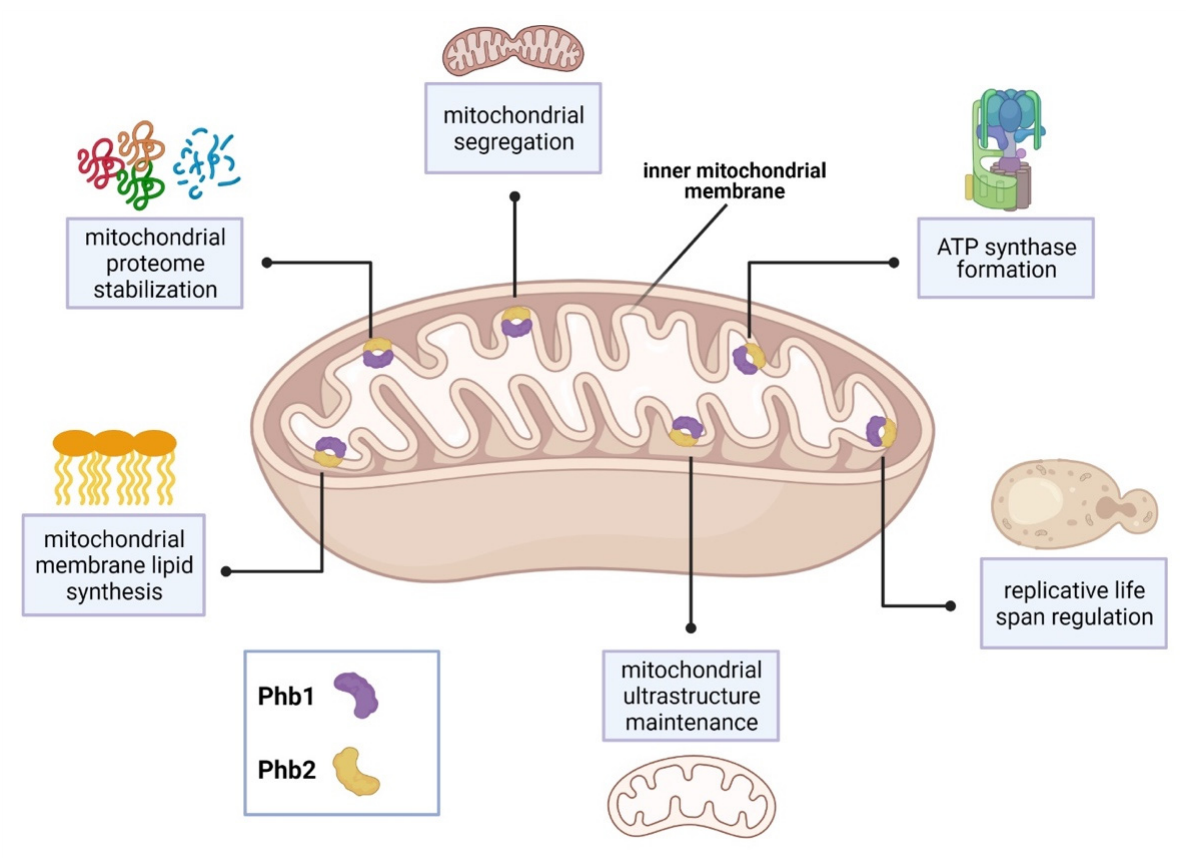
SPFH protein distribution and functions in *S. cerevisiae*. Phb1 and Phb2 form ring-shaped complexes that localize to the inner mitochondrial membrane. Known mitochondrial functions associated with the Phb1/Phb2 complex are depicted in the blue boxes. Figure adapted from references [9,34,35,36,37,39] and created with Biorender.com.

Synthetic genetic arrays using a *phb1Δ* mutant strain identified 35 genes that are required for viability or normal growth [36]. Interestingly, 31 of these genes encode mitochondrial proteins. 19 of these genes were associated with respiratory chain assembly and maintenance of mitochondrial structure. Major *PHB1* genetic partners include *YTA10*, *YTA11*, and *YME1* [36]. These genes encode proteins which belong to the conserved, ATP-dependent mitochondrial *m*-AAA protease family, which maintain the mitochondrial proteome [41]. Other *PHB1* genetic partners include the cytochrome c complex subunit-encoding genes, *COX6* and *COX24* [36]. In addition, 8 genes are required for the synthesis of the mitochondrial membrane lipids, cardiolipin and phosphatidylethanolamine. These partners include the highly conserved genes, *UPS1* and *UPS2* [36]. Lastly, prohibitin function and localization was associated with the presence of the yeast [*PSI^+^*] prion. Proteomic analysis revealed that aberrant mitochondrial function observed in [*PSI^+^*] prion yeast strains was caused, in part, by Phb1 mislocalization in the cytoplasm [35]. See Figure 1 for a summary of SPFH function in *S. cerevisiae*.

SPFH function has also been characterized in other non-pathogenic fungi. In the fission yeast *Schizosaccharomyces pombe*, Phb1 and Phb2 localize to the mitochondria [33]. Overexpression or deletion of the *phb2* gene caused resistance to various antifungal drugs including terbinafine, fluconazole, amphotericin B, and clotrimazole [33]. Moreover, increased production of intracellular nitric oxide and reactive oxygen species were observed in Phb2 overexpression or deletion strains [33]. In contrast, only a *Δphb1* deletion strain was resistant to antifungal drugs [33]. Additional genetic evidence showed that mitochondrial dysfunction caused by *phb2* deletion and overexpression activated the oxidative stress response transcriptional regulator, Pap1, thus linking prohibitins to stress response signaling [33]. Paradoxically, *S. cerevisiae phb2Δ* mutants were sensitive to fluconazole, amphotericin B, and clotrimazole, highlighting the differences of SPFH protein function in different yeast species [33]. However, the basis of this phenotype is unknown.

Matrix-assisted laser desorption ionization-time of flight mass spectrometry (MALDI-TOF-MS) analyses on mitochondrial extracts identified three SPFH proteins (Phb1, Phb2 and Slp2) in the filamentous fungus, *Neurospora crassa* [38]. Consistent with the structural dynamics of prohibitins from mammals, nematodes, and yeast, *N. crassa* Phb1 and Phb2 localized to the inner mitochondrial membrane and formed large membrane complexes of various sizes. Notably, one high molecular weight prohibitin complex co-migrated with *m*-AAA protease MAP-1. This suggests that *m*-AAA proteins may physically interact with prohibitins in *N. crassa*, similar to observations with *S. cerevisiae* prohibitins [38]. Moreover, the stomatin, Slp2, was found to co-migrate in a high molecular weight complex with the *N. crassa i*-MMM protease homolog, IAP-1, suggesting that Slp2 and IAP-1 physically interact in the inner mitochondrial membrane [38]. Taken together, these findings demonstrate the importance of SPFH protein function in mitochondrial ultrastructure, respiratory function, and antifungal drug resistance.

## 3. SPFH Protein Function in Pathogenic Fungi

In fungal pathogens, mitochondria are required for virulence determinants including morphogenesis, drug susceptibility, cell wall biogenesis, and biofilm formation [42,43,44]. The knowledge underlying the molecular and cellular aspects of mitochondrial function in human pathogenic fungi is based primarily on studies in *Candida albicans*. *C. albicans* resides on mucosal tissue in the oral cavity and genitourinary and gastrointestinal tracts in a harmless commensal state [45]. Under permissive conditions, such as a change in host immunity, *C. albicans* causes superficial vaginal or oral mucosal infections. Disseminated invasive candidiasis is a major cause of morbidity and mortality for immunocompromised patients [46,47,48]. Mitochondrial function is critical for *C. albicans* commensalism and virulence [42]. Indeed, *C. albicans* cells treated with respiratory inhibitors display aberrant cell wall structure and increased macrophage recognition [49]. Moreover, mutations to fungal-specific mitochondrial genes, such as *GOA1, NUO3, NUO4,* and *GEM1*, attenuate virulence [50,51,52,53]. Genome-wide transcriptional profiling revealed that genes encoding proteins with mitochondrial functions are significantly upregulated following cell wall damage or osmotic stress [54,55,56]. The molecular details underlying *C. albicans* respiration have been reviewed [42,45,57,58]. The expanded role of the mitochondria in growth, stress adaptation, and virulence underscores the need to study all aspects of mitochondrial function. Therefore, SPFH proteins are excellent candidates to broaden our understanding of *C. albicans* mitochondrial dynamics.

The *C. albicans* genome includes five SPFH family members: *PHB1*, *PHB2*, *PHB12*, *SLP2*, and *SLP3* (stomatin-like protein 3) [59]. We were the first group to identify a role for SPFH proteins in *C. albicans*. We found that *SLP3* transcription and protein localization significantly increased following treatment with oxidative, osmotic, cell wall, or plasma membrane stress agents, categorizing *SLP3* as a general stress response gene [5,55,56]. Slp3p formed visible puncta along the plasma membrane similar to mammalian stomatin complexes when viewed using fluorescence microscopy [5,60]. Slp3 plasma membrane localization was also confirmed via liquid chromatography-mass spectrometry (LC-MS/MS) and MALDI-TOF analysis on *C. albicans* plasma membrane extracts [6]. Interestingly, we also observed Slp3 localization at the vacuolar lumen; however, the basis for this result is unknown [5]. *SLP3* transcription was significantly downregulated in cells undergoing the yeast-to-hyphae transition [61]. In concordance with this observation, Slp3 plasma membrane and vacuolar localization was absent in hyphal cells, categorizing Slp3 as a yeast-phase specific protein. [5]. A *slp3Δ/slp3Δ* homozygous mutant strain did not show a growth defect under standard growth conditions or when exposed to a variety of environmental stress conditions or antifungal drugs [5]. Moreover, the *slp3Δ/slp3Δ* mutant did not display any apparent cell structure abnormality, organelle malfunction, or ion transport defect [5].

SPFH protein overproduction in mammals, nematodes, yeast, and mice causes a broad array of phenotypes, including drug resistance, aging, apoptosis, and tumorigenesis [27,28,62]. Consistent with these observations, we found that *C. albicans* Slp3 overproduction severely disrupted mitochondrial membrane potential and triggered apoptotic-like death specifically following prolonged exposure to oxidative stress [5]. Moreover, Slp3 overproduction in hyphal cells caused aberrant filament structure [5].

**Figure 2:**
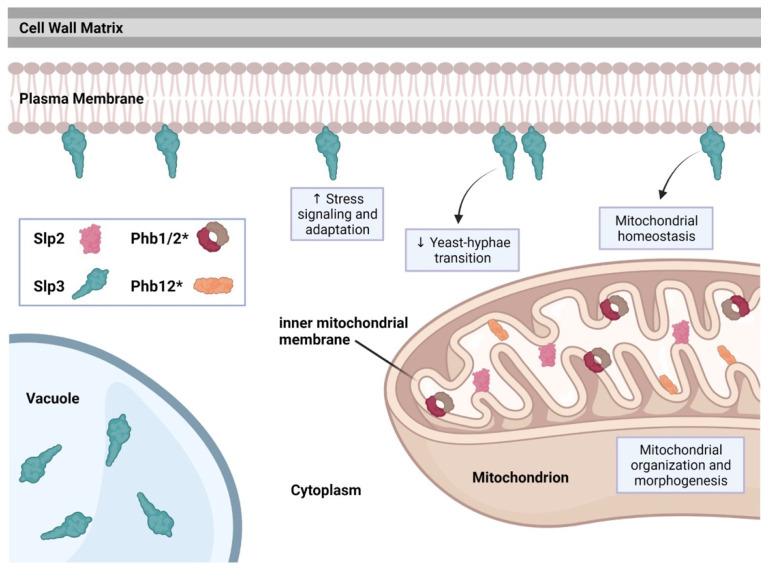
SPFH protein distribution and function in *C. albicans*. Illustration depicting the cellular localization of SPFH family members Slp2, Slp3, Phb1, Phb2, and Phb12. Asterisks (*) beside Phb1, Phb2, and Phb12 denote that cellular localization of these proteins are only predicted and have not been experimentally confirmed. Biochemical and cytological analyses demonstrated that Slp3 localizes to the plasma membrane and vacuole, and Slp2 localizes to the mitochondria. Known functions associated with Slp3 are depicted in the blue boxes. Slp3 production is increased in response to environmental stress (boxed upward pointing arrow). Slp3 production is decreased in the yeast-to-hyphae transition (boxed downward pointing arrow). Figure adapted from references [5,6] and our unpublished findings and created with Biorender.com.

*C. albicans* Slp2, Phb1, Phb2, and Phb12 each contain a putative mitochondrial localization signal motif (our preliminary findings). We observed Slp2 mitochondrial localization (our preliminary findings), suggesting that the mitochondrial functions for prohibitins and Slp2 may be conserved. See Figure 2 for a summary of *C. albicans* SPFH protein function.

**Figure 3:**
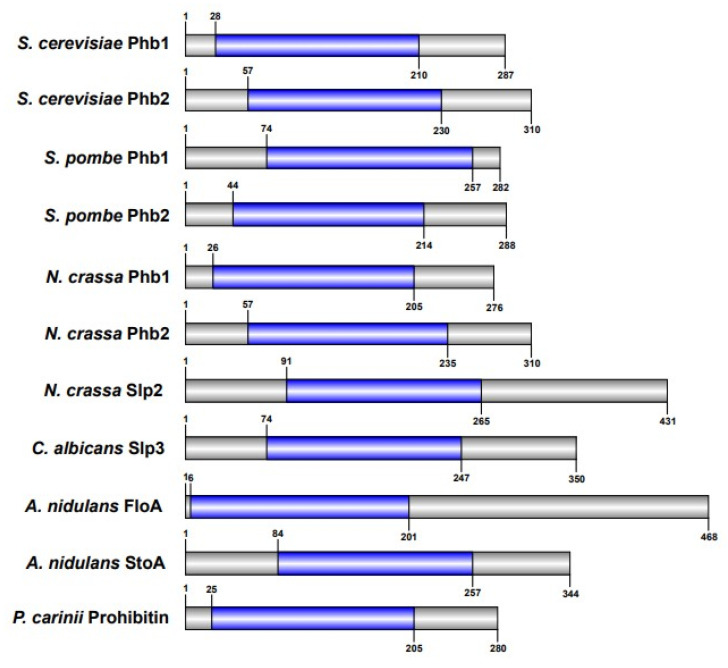
Schematic of fungal SPFH proteins. SPFH proteins that have been experimentally validated in fungi are depicted. Sequences for each protein were obtained from the *Saccharomyces* Genome Database, PomBase, *Candida* Genome Database, *Aspergillus* Genome Database, or UniProt [59,65,66]. SPFH/Band 7 domains (blue) were identified using InterProScan [67]. Schematics were constructed and drawn to scale using Illustrator for Biological Sequences [68].

Other pathogenic fungi where SPFH protein function has been investigated include *Aspergillus nidulans* and *Pneumocystis carinii*. For both species, SPFH proteins were not associated with mitochondrial function. In *A. nidulans*, the flotillin, FloA, localized to the plasma membrane and mediates formation of plasma membrane sterol-rich domains. The stomatin, StoA, localized to the plasma membrane and endosomal/vacuolar-like structures and is required for hyphal polarized growth [63]. Heterologous expression of *P. carinii* prohibitin in human fibroblasts caused cell cycle arrest, suggesting a role for prohibitin in regulating proliferation and development [64]. Collectively, these findings highlight the expanded role of SPFH proteins in growth, filamentation, mitochondrial function, and stress signaling in pathogenic fungi. See Table 1 and Figure 3 for a summary and schematic of SPFH proteins with known functions in various fungal species.

## 4. Perspectives and Future Directions

It is critical that we advance our understanding of mitochondrial function in fungi to facilitate the development of new approaches for antifungal interventions [69]. The efficacy of antifungal drugs is limited due to mammalian tissue toxicity, parenteral formulations, and emerging drug resistant species [45,70]. Mitochondria are a premier pharmacological target in candidiasis treatment. The frequently prescribed antifungal drug, fluconazole, targets the lipid-synthesizing mitochondrial protein Erg11 [71].

The N- and C-terminal primary sequences between *C. albicans* and human SPFH proteins are highly divergent [5]; therefore, these regions can be potentially exploited in the development of novel antifungal therapeutics. For example, our findings that Slp3 overproduction disrupts *C. albicans* filamentation and depolarizes mitochondria can be used as a basis to develop a SPFH-derived antifungal strategy. In support of this idea, the therapeutic potential of targeting SPFH proteins have been recently demonstrated in bacteria, worms, and mammals [72]. The flavaglines class of natural chemical compounds inhibits mitochondrial PHB2 function, blocking mitophagy in human cancer cells [19]. Moreover, the small molecule synthetic compounds, OB-1 and OB-2 (oligomerization blockers 1 and 2), inhibit STOML3-dependent mechanosensation, thus alleviating painful diabetic neuropathy in mice [60].

Despite the progress made in revealing the structural characteristics and biochemical mechanisms governing SPFH protein function in model eukaryotes, we still do not know the function, physical and genetic targets, and mechanism-of-action for SPFH family members in pathogenic fungi. For example, the impact of *C. albicans* Slp3 on mitochondrial function suggests that SPFH proteins may mediate direct interactions between the plasma membrane or vacuole with the mitochondria. In *S. cerevisiae*, mitochondria directly associate with the plasma membrane and organelles such as the vacuole or endoplasmic reticulum to mediate lipid metabolism and mitochondrial biogenesis [73]. SPFH proteins have not yet been implicated in those interactions. In addition, the absence of an apparent phenotype in a *slp3Δ/slp3Δ* mutant suggests there may be functional redundancy among *C. albicans* SPFH proteins.

Thus, a comprehensive analysis for SPFH protein function in *C. albicans* and other fungal pathogens will require a proteomic and genetic approach along with a cost-effective in vivo infection assay. The recent development of optimized CRISPR-Cas9 genome editing methods in *C. albicans* [74] will facilitate the development of yeast strains containing mutations to multiple SPFH genes or SPFH genes and candidate SPFH protein targets to investigate genetic interactions. Hetero-oligomeric complexes with SPFH proteins and various transporter proteins as well as among SPFH stomatins and prohibitins have been observed in humans [10,14,75]. Co-Interacting Protein Identification Technology (Co-PIT) [76], and LC-MS/MS analyses can be used to determine the constituents of SPFH protein complexes in *C. albicans*.

Finally, the invertebrate planarian, *Schmidtea mediterranea*, has recently been shown to be an excellent host to study *C. albicans* pathogenesis in vivo [77]. The simple planarian anatomical design allows visualization of all phases of *C. albicans* infection, such as adherence, yeast-to-hyphae transition, and invasive growth in a cost-effective and time-saving manner [77]. To determine the role of SPFH proteins in *C. albicans* virulence, SPFH mutant and overexpressing strains can be analyzed in infection assays. Collectively, such findings will characterize the SPFH family in the context of a critical area in *C*. *albicans* biology: The molecular framework underlying mitochondrial function. In addition, the knowledge gained from *C. albicans* SPFH analysis will provide a detailed model for SPFH function in other fungal pathogens, such as *Candida auris* and *Candida glabrata*.

## Figures and Tables

**Table 1:** SPFH proteins with known functions in fungi. SPFH protein localization and function for each fungal species listed have been experimentally confirmed in the referenced literature.

Protein	Localization	Function	References
***S. cerevisiae***			
Phb1 and Phb2	inner mitochondrial membrane, Phb1-Phb2 complex	regulation of mitochondrial ultrastructure and segregation, mitochondrial protein stabilization, regulation of replicative life span, regulation of mitochondrial membrane lipid synthesis, involvement in ATP synthase formation	[34,35,36,37,39,40]
***S. pombe***			
Phb1	mitochondria	multi-drug resistance	[33]
Phb2	mitochondria	multi-drug resistance, oxidative stress signaling	[33]
***C. albicans***			
Slp3	plasma membrane, vacuolar lumen	yeast-specific general stress response signaling, involved in maintenance of mitochondrial membrane integrity	[5,6]
***N. crassa***			
Slp2	mitochondria	interactions with *i*-MMM protein IAP-1	[38]
Phb1 and Phb2	inner mitochondrial membrane, Phb1-Phb2 complex	interactions with *m*-AAA protease MAP-1	[38]
***A. nidulans***			
FloA	plasma membrane	formation of sterol-rich domains in plasma membrane	[63]
StoA	plasma membrane, endosome/vacuole	polarized hyphal growth	[63]
***P. carinii***			
Prohibitin	inner mitochondrial membrane (predicted)	regulation of cell proliferation and development	[64]
